# Supplementary material for: Associations between the Home Environment, Feeding Practices and Children’s Intakes of Fruit, Vegetables and Confectionary/Sugar-Sweetened Beverages
Source: Int J Environ Res Public Health. 2020 Jul 5;17(13):4837. doi: 10.3390/ijerph17134837 (PMC7370037; doi:10.3390/ijerph17134837)
Supplement: Supplementary file 1 [file ijerph-17-04837-s001.zip › Figure S1.docx]

*^1^*Analysis using MANOVA after controlling for the cofounding factor, parents’ nationality.

**Figure S1.** Parental control feeding practice by parents' education level
