# Supplementary material for: Associations between the Home Environment, Feeding Practices and Children’s Intakes of Fruit, Vegetables and Confectionary/Sugar-Sweetened Beverages
Source: Int J Environ Res Public Health. 2020 Jul 5;17(13):4837. doi: 10.3390/ijerph17134837 (PMC7370037; doi:10.3390/ijerph17134837)
Supplement: Supplementary file 1 [file ijerph-17-04837-s001.zip › Table S1.docx]

| **Table S1.** Participant and home environmental characteristics resulting as significant after bivariate analysis with children's fruit intake | | | | | | |
| --- | --- | --- | --- | --- | --- | --- |
|  |  | Fruit | | | |  |
|  |  | **≥ 1 serving a day** | | **< 1 serving a day** | |  |
| **Characteristics** | | ***n^1^*** | **%** | ***n*** | **%** | *p** |
| Education level | Higher | 149 | 63.4 | 39 | 40.2 | <0.001 |
|  | Lower | 86 | 36.6 | 58 | 59.8 |  |
| Parents’ BMI | Normal weight | 143 | 66.5 | 44 | 50.6 | 0.010 |
|  | Overweight/obese | 72 | 33.5 | 43 | 49.4 |  |
| Parents’ fruit intake | ≥ 3 servings a day | 64 | 27.2 | 13 | 13.4 | 0.007 |
|  | < 3 servings a day | 171 | 72.8 | 84 | 86.6 |  |
| Parents’ vegetable intake | ≥ 3 servings a day | 88 | 37.4 | 21 | 21.6 | 0.005 |
|  | < 3 servings a day | 147 | 62.6 | 76 | 78.4 |  |
| Parents’ SSB intake | ≥ 1 time per week | 43 | 18.3 | 34 | 35.1 | 0.001 |
|  | < 1 time per week | 192 | 81.7 | 63 | 64.9 |  |
| Children’s daily television viewing | < 1 hour daily | 115 | 48.9 | 31 | 32 | 0.005 |
|  | ≥ 1 hour daily | 120 | 51.1 | 66 | 68 |  |
| Parents set rules about television viewing | No | 41 | 17.5 | 26 | 27.1 | 0.050 |
|  |  |  |  |  |  |  |
| Parents allow snacks to be eaten in front of television | Frequently | 177 | 75.6 | 83 | 87.4 | 0.018 |
|  | Rarely/never | 57 | 24.4 | 12 | 12.6 |  |
|  |  | **mean^2^** | **SD** | **mean** | **SD** |  |
| Parental control feeding practice | Pressure | 2.82 | 1.0 | 3.18 | 1.0 | 0.003 |
|  | Monitoring | 4.34 | 0.9 | 4.08 | 1.0 | 0.026 |
|  |  | **median ^3^** | **IQR** | **median** | **IQR** |  |
| Home food availability | Number of fruit types | 5.0 | 4.0 - 6.0 | 4.0 | 3.0 - 5.0 | <0.001 |
|  | Number of vegetable types | 7.0 | 5.0 - 9.0 | 6.0 | 4.2 - 8.0 | 0.007 |
| Home food accessibility (*child can reach food without help)* | | ***n^1^*** | **%** | ***n*** | **%** |  |
| Sweet snacks | Yes | 27 | 12.5 | 23 | 24.2 | 0.004 |
| SSBs | Yes | 55 | 24.8 | 37 | 38.9 | 0.005 |
| IQR: Interquartile range, SD: Standard deviation. **p* < 0.05 was significant; ^1^ Association between categorical variables assessed using the chi-squared test with Yates’ Continuity Correction for 2x2 contingency tables; ^2^Association between normally distributed continuous data assessed using an Independent Samples t-test; ^3^Association between non-normally distributed continuous data assessed using a Mann-Whitney U test | | | | | | |
