# Supplementary material for: Associations between the Home Environment, Feeding Practices and Children’s Intakes of Fruit, Vegetables and Confectionary/Sugar-Sweetened Beverages
Source: Int J Environ Res Public Health. 2020 Jul 5;17(13):4837. doi: 10.3390/ijerph17134837 (PMC7370037; doi:10.3390/ijerph17134837)
Supplement: Supplementary file 1 [file ijerph-17-04837-s001.zip › Table S2.docx]

| **Table S2.** Participant and home environmental characteristics resulting as significant after bivariate analysis with children's vegetables intake | | | | | | |
| --- | --- | --- | --- | --- | --- | --- |
|  |  | Vegetables | | | |  |
|  |  | **≥ 1 serving a day** | | **< 1 serving a day** | |  |
| **Characteristics** | | ***n^1^*** | **%** | ***n*** | **%** | *p** |
| Education level | Higher | 90 | 69.8 | 98 | 48.3 | <0.001 |
|  | Lower | 39 | 30.2 | 105 | 51.7 |  |
| Parents’ BMI | Normal weight | 83 | 69.7 | 104 | 56.8 | 0.010 |
|  | Overweight/obese | 36 | 69.7 | 79 | 43.2 |  |
| Time weaning established | < 17 weeks | 24 | 19.2 | 56 | 29.2 | 0.046 |
|  | ≥ 17 weeks | 101 | 80.8 | 136 | 70.8 |  |
| Parents’ vegetable intake | ≥ 3 servings a day | 65 | 50.4 | 44 | 21.7 | 0.005 |
|  | < 3 servings a day | 64 | 49.6 | 159 | 78.3 |  |
| Prepare meals at home | < 5 times per week | 17 | 13.2 | 44 | 21.7 | 0.050 |
|  | ≥ 5 times per week | 112 | 86.8 | 159 | 78.3 |  |
| Eat main meal with one or both parents | < 5 times per week | 32 | 25.2 | 75 | 37.1 | 0.025 |
|  | ≥ 5 times per week | 95 | 74.8 | 127 | 62.9 |  |
| Microwavable or quick-cook frozen foods consumed | Frequently | 32 | 25 | 76 | 37.8 | 0.016 |
|  | Rarely/never | 96 | 75 | 125 | 62.2 |  |
| Takeaway food which the child also eats | Frequently | 58 | 45.3 | 113 | 56.5 | 0.048 |
|  | Rarely/never | 70 | 54.7 | 87 | 43.5 |  |
| Children’s daily television viewing | < 1 hour daily | 77 | 59.7 | 69 | 34 | 0.001 |
|  | ≥ 1 hour daily | 52 | 40.3 | 134 | 66 |  |
| Parents set rules about television viewing | No | 17 | 13.2 | 50 | 24.9 | <0.011 |
|  |  |  |  |  |  |  |
| Parents allow meal to be eaten in front of television | Frequently | 50 | 39.1 | 123 | 60.9 | <0.001 |
|  | Rarely/never | 78 | 60.9 | 79 | 39.1 |  |
| Parents allow snacks to be eaten in front of television | Frequently | 80 | 62.5 | 180 | 89.6 | <0.001 |
|  | Rarely/never | 48 | 37.5 | 21 | 10.4 |  |
|  |  | **mean^2^** | **SD** | **mean** | **SD** |  |
| Parental control feeding practice | Pressure | 2.61 | 0.9 | 3.12 | 1.0 | <0.001 |
|  | Monitoring | 4.16 | 0.9 | 4.42 | 0.9 | 0.014 |
|  |  | **median ^3^** | **IQR** | **median** | **IQR** |  |
| Home food availability | Fruit types | 5.0 | 4.0 - 6.0 | 4.0 | 3.0 - 5.0 | <0.001 |
|  | Vegetable types | 7.0 | 6.0 - 9.0 | 6.0 | 5.0 - 8.0 | <0.001 |
|  | Sweet snack types | 2.0 | 1.0 - 3.0 | 3.0 | 2.0 - 3.7 | 0.032 |
|  | SSB types | 1.0 | 0.0 - 1.0 | 1.0 | 0.0 - 2.0 | 0.007 |
|  | Savoury snack types | 1.0 | 0.0 - 2.0 | 2.0 | 1.0 - 3.0 | 0.004 |
| Home food accessibility (*child can reach food without help)* | | ***n^1^*** | **%** | ***n*** | **%** |  |
| Fruit | Yes | 103 | 81.1 | 139 | 69.5 | 0.020 |
| Sweet snacks | Yes | 12 | 9.3 | 38 | 18.8 | 0.018 |
| IQR: Interquartile range, SD: Standard deviation. **p* < 0.05 was significant; ^1^ Association between categorical variables assessed using the chi-squared test with Yates’ Continuity Correction for 2x2 contingency tables; ^2^Association between normally distributed continuous data assessed using an Independent Samples t-test; ^3^Association between non-normally distributed continuous data assessed using a Mann-Whitney U test | | | | | | |
