# Supplementary material for: Associations between the Home Environment, Feeding Practices and Children’s Intakes of Fruit, Vegetables and Confectionary/Sugar-Sweetened Beverages
Source: Int J Environ Res Public Health. 2020 Jul 5;17(13):4837. doi: 10.3390/ijerph17134837 (PMC7370037; doi:10.3390/ijerph17134837)
Supplement: Supplementary file 1 [file ijerph-17-04837-s001.zip › Table S3.docx]

| **Table S3.** Participants and home environmental characteristics resulting as significant after bivariate analysis with children's confectionary/SSB intakes | | | | | | |
| --- | --- | --- | --- | --- | --- | --- |
|  |  | **Confectionary/SSBs** | | | |  |
|  |  | **≥ 1 serving a day** | | **< 1 serving a day** | |  |
| **Characteristics** | | ***n^1^*** | **%** | ***n*** | **%** | *p** |
| Nationality | Irish | 169 | 70.1 | 53 | 58.2 | 0.040 |
|  | Not Irish | 72 | 29.9 | 38 | 41.8 |  |
| Education level | Higher | 122 | 50.6 | 66 | 72.5 | <0.001 |
|  | Lower | 119 | 49.4 | 25 | 27.5 |  |
| Parents’ BMI | Normal weight | 124 | 57.4 | 63 | 73.3 | 0.010 |
|  | Overweight/obese | 92 | 42.6 | 23 | 26.7 |  |
| Age weaning established | < 17 weeks | 72 | 31.6 | 8 | 9 | 0.008 |
|  | ≥ 17 weeks | 156 | 68.4 | 81 | 91 |  |
| Parents’ vegetable intake | ≥ 3 servings a day | 69 | 28.6 | 40 | 44 | 0.008 |
|  | < 3 servings a day | 172 | 71.4 | 51 | 56 |  |
| Parents’ confectionary/savoury snack intakes | ≥ 3 times per week | 128 | 53.1 | 31 | 34.1 | 0.002 |
|  | < 3 times per week | 113 | 46.9 | 60 | 65.9 |  |
| Parents’ SSB intakes | ≥ 1 time per week | 70 | 29 | 7 | 7.7 | <0.001 |
|  | < 1 time per week | 171 | 71 | 84 | 92.3 |  |
| Microwavable or quick-cook frozen foods consumed | Frequently | 87 | 36.4 | 21 | 23.3 | 0.024 |
|  | Rarely/never | 152 | 63.6 | 69 | 76.7 |  |
| Takeaway food which the child also eats | Frequently | 140 | 58.8 | 31 | 34.4 | <0.001 |
|  | Rarely/never | 98 | 41.2 | 59 | 65.6 |  |
| Children’s daily television viewing | < 1 hour daily | 93 | 38.6 | 53 | 58.2 | 0.001 |
|  | ≥ 1 hour daily | 148 | 61.4 | 38 | 41.8 |  |
| Parents allow snack to be eaten in front of television | Frequently | 204 | 85.4 | 56 | 62.2 | <0.001 |
|  | Rarely/never | 35 | 14.6 | 34 | 37.8 |  |
|  |  | **median ^2^** | **IQR** | **median** | **IQR** |  |
| Home food availability | Vegetable types | 7.0 | 5.0 - 8.0 | 7.0 | 6.0 - 9.0 | 0.001 |
|  | Sweet snack types | 3.0 | 2.0 - 4.0 | 2.0 | 1.0 - 3.0 | <0.001 |
|  | SSB types | 1.0 | 0.0 - 2.0 | 0.0 | 0.0 - 1.0 | <0.001 |
|  | Savoury snack types | 2.0 | 1.0 - 2.0 | 1.0 | 0.0 - 2.0 | 0.001 |
| Home food accessibility *(child can reach food without help)* | | ***n^1^*** | **%** | ***n*** | **%** |  |
| Vegetables | No | 196 | 84.5 | 60 | 70.6 | 0.005 |
| Sweet snack | Yes | 43 | 18.6 | 7 | 8.8 | 0.038 |
| SSBs | Yes | 77 | 33 | 15 | 17.9 | 0.009 |
| IQR: Interquartile range; **p* < 0.05 was significant; ^1^ Association between categorical variables assessed using the chi-squared test with Yates’ Continuity Correction for 2x2 contingency tables; ^2^Association between non-normally distributed continuous data assessed using a Mann-Whitney U test | | | | | | |
